# New Technologies and Materials in Oral Health and Dental Care of Pediatric Dentistry

**DOI:** 10.3390/children12101310

**Published:** 2025-09-29

**Authors:** Giuseppe Minervini

**Affiliations:** 1 Multidisciplinary Department of Medical-Surgical and Dental Specialties, University of Campania ‘Luigi Vanvitelli’, 80138 Naples, Italy; giuseppe.minervini@unicampania.it; 2 Saveetha Dental College and Hospitals, Saveetha Institute of Medical and Technical Sciences (SIMATS), Saveetha University, Chennai 600077, India

Pediatric dentistry is undergoing an exciting evolution. From smarter diagnostic tools to more thoughtful behavior management strategies, the field is expanding rapidly in response to the complex and diverse needs of children and adolescents. This Special Issue of Children reflects this transformation by featuring a wide range of innovative studies and insightful reviews that address both long-standing and emerging challenges in pediatric oral healthcare.

The importance of durable, esthetic, and biologically compatible restorative options in children cannot be overstated. In a multicenter randomized trial, Lee et al. demonstrated the clinical viability of three-dimensional (3D)-printed resin crowns for restoring primary molars [1]. While stainless steel crowns continue to outperform in terms of survival, resin crowns show promising results in esthetics and repairability—highlighting the potential of 3D printing as a transformative tool in pediatric prosthodontics [2].

Laser activation of sodium hypochlorite irrigation in primary teeth significantly improves disinfection outcomes. The current literature shows that diode laser-activated NaOCl gel eradicates *Enterococcus faecalis* in primary canals, offering a potential protocol to enhance microbial decontamination while minimizing the need for more invasive mechanical instrumentation [3]. Also, during caries removal, the Er:YAG laser has shown superior preservation of sound dental tissues and reduced postoperative discomfort compared to the traditional dental turbine tool [4].

Technological advances are reshaping orthodontic anchorage techniques. Vasoglou et al. described a digitally assisted technique using mini-implants and 3D-printed surgical guides to manage bimaxillary dentoalveolar protrusion in adolescents—avoiding extractions and delivering notable facial esthetic improvements [5].

Digital planning is also enhancing outcomes in pediatric maxillofacial surgery. A fully digital workflow—including CT-based virtual planning and 3D-printed surgical guides—enables precise and safe treatment of mandibular fractures in children, showing excellent agreement between preoperative planning and postoperative outcomes [6].

Artificial intelligence (AI) is steadily gaining ground in pediatric dental care. Mladenovic et al. reported the successful use of AI-based platforms such as Diagnocat for the detection and management of non-syndromic supernumerary teeth, facilitating earlier and more accurate intervention [7]. In parallel, Acharya et al. reviewed AI’s potential in real-time behavioral management strategies, which is particularly useful for managing anxious or uncooperative pediatric patients [8].

For children requiring extensive or complex treatment, particularly those with special needs, pharmacological sedation is a critical component of care. Inchingolo et al. conducted a systematic review exploring the efficacy and safety of oral, intranasal, and inhalational sedation techniques, offering updated insights into multimodal sedation protocols [9]. Martínez Pérez et al. emphasized the value of behavioral desensitization protocols for children with autism spectrum disorders [10]. The findings demonstrated that shorter latency between desensitization sessions and the actual dental appointment significantly improved patient cooperation, reinforcing the need for multidisciplinary collaboration between parents, educators, and dental teams [11].

The psychosocial dimension of pediatric dentistry continues to gain relevance. A recent study investigated the impact of intermaxillary elastics in clear aligner therapy, revealing a notable association with increased pain and psychosocial distress. The findings suggest that orthodontic mechanics—though clinically effective—can heighten temporomandibular symptoms if not carefully managed, emphasizing the need for behavioral assessments alongside biomechanical planning [12].

Meanwhile, digital platforms are increasingly influencing how caregivers access and interpret health information [13,14]. An analysis of online bruxism-related content found that much of it has poor readability and questionable quality, raising concerns about misinformation among parents seeking guidance online [15]. Another study further corroborated this issue through a review of YouTube videos on pediatric oral lesions. While online media are widely consulted, the inconsistent quality highlights an urgent need for clinician-led, accessible, and evidence-based content [16,17]. These insights collectively stress that successful pediatric care must extend beyond clinical expertise to include communication strategies that guide families through the digital landscape and consider children’s behavioral well-being.

Pediatric dentistry is a field where innovation and empathy go hand in hand [18,19]. Technologies and strategies—from AI-assisted diagnostics to laser-enhanced endodontics and digitally guided orthodontics—underscore how science and care can converge to improve pediatric oral health outcomes [20,21]. As these innovations continue to evolve, it is our collective responsibility to ensure they are used ethically and equitably—empowering young patients, supporting families, and fostering healthier, more confident smiles.
